# Alteration of *De Novo* Glucose Production Contributes to Fasting Hypoglycaemia in Fyn Deficient Mice

**DOI:** 10.1371/journal.pone.0081866

**Published:** 2013-11-28

**Authors:** Yingjuan Yang, Elena Tarabra, Gong-She Yang, Bhavapriya Vaitheesvaran, Gustavo Palacios, Irwin J. Kurland, Jeffrey E. Pessin, Claire C. Bastie

**Affiliations:** 1 Laboratory of Animal Fat Deposition and Muscle Development, College of Animal Science and Technology, Northwest A&F University, Yangling, Shaanxi, People’s Republic of China; 2 Department of Medicine, Albert Einstein College of Medicine, Bronx, New York, United States of America; 3 Department of Chemical Biology and Therapeutics, St. Jude Children’s Research Hospital, Memphis, Tennessee, United States of America; 4 Department of Molecular Pharmacology, Albert Einstein College of Medicine, Bronx, New York, United States of America; 5 Division of Metabolic and Vascular Health, Warwick Medical School, University of Warwick, Coventry, United Kingdom; University of Minnesota - Twin Cities, United States of America

## Abstract

Previous studies have demonstrated that glucose disposal is increased in the Fyn knockout (FynKO) mice due to increased insulin sensitivity. FynKO mice also display fasting hypoglycaemia despite decreased insulin levels, which suggested that hepatic glucose production was unable to compensate for the increased basal glucose utilization. The present study investigates the basis for the reduction in plasma glucose levels and the reduced ability for the liver to produce glucose in response to gluconeogenic substrates. FynKO mice had a 5-fold reduction in phosphoenolpyruvate carboxykinase (PEPCK) gene and protein expression and a marked reduction in pyruvate, pyruvate/lactate-stimulated glucose output. Remarkably, *de novo* glucose production was also blunted using gluconeogenic substrates that bypass the PEPCK step. Impaired conversion of glycerol to glucose was observed in both glycerol tolerance test and determination of the conversion of ^13^C-glycerol to glucose in the fasted state. α-glycerol phosphate levels were reduced but glycerol kinase protein expression levels were not changed. Fructose-driven glucose production was also diminished without alteration of fructokinase expression levels. The normal levels of dihydroxyacetone phosphate and glyceraldehyde-3-phosphate observed in the FynKO liver extracts suggested normal triose kinase function. Fructose-bisphosphate aldolase (aldolase) mRNA or protein levels were normal in the Fyn-deficient livers, however, there was a large reduction in liver fructose-6-phosphate (30-fold) and fructose-1,6-bisphosphate (7-fold) levels as well as a reduction in glucose-6-phosphate (2-fold) levels. These data suggest a mechanistic defect in the allosteric regulation of aldolase activity.

## Introduction

The regulation of glucose homeostasis is a complex integrative response between multiple tissues that dynamically respond to metabolic and nutritional states. Classically, glucose metabolism is predominantly controlled through the counter-regulatory actions of insulin and glucagon. In the fed state, insulin signalling in peripheral tissues (skeletal muscle and adipose tissue) increases glucose uptake and in the liver, insulin drives glycogen synthesis and suppresses hepatic glucose production [1]. In the fasted state, glucagon stimulates hepatic glucose release into the circulation to ensure a relatively constant glucose supply for peripheral tissues [2]. As such, circulating blood glucose levels are tightly regulated to remain constant irrespective of dietary nutritional input. Net hepatic glucose release occurs when dietary carbohydrates are unavailable resulting from two tightly regulated pathways: glycogenolysis and *de novo* synthesis of glucose (gluconeogenesis). Although the exact contribution of each process to glucose production is still controversial, gluconeogenesis has a greater importance for prolonged fasting periods in mice, since glycogen stores are likely to be nearly depleted after the first few hours following food withdrawal. 

To ensure that glucose production matches the whole-body requirements, gluconeogenesis must be tightly regulated. This physiologic regulation fails in diabetes and obesity states with concomitant exacerbation of glucagon responsiveness and defective insulin-driven suppression of hepatic glucose output [3–5]. For this reason, mechanisms by which *de novo* glucose production is controlled have been intensively investigated and numerous studies have focused on the transcriptional control of genes mediating liver glucose metabolism. Classically, expression levels of phosphoenolpyruvate carboxykinase (PEPCK) are considered control point for liver gluconeogenesis [6,7]. However, the allosteric regulation of glycolysis, glycogen synthesis, glycogenolysis and gluconeogenesis remains the primary acute mechanism responsible for controlling the directional carbon flux between catabolism and anabolism.

During our initial investigation on the role of Fyn kinase in integrative metabolism, we demonstrated that whole-body Fyn deficiency resulted in lean animals with reduced adiposity and lower circulating and intra-tissue fatty acids and triglycerides [8]. In addition, although glucose disposal was also increased in FynKO mice, fasting blood glucose levels were approximately 30% reduced compared to control mice after a 16-hour fasting period, which suggested that hepatic glucose production was not compensating for the increased peripheral tissue glucose demand. 

In this study, we now demonstrate that the liver of the FynKO mice displays reduced glucose production from three-carbon gluconeogenic precursors, i.e., pyruvate, lactate and glycerol as well as from the six-carbon sugar fructose. This suggests a defect in the fructose-bisphosphate aldolase, the enzyme responsible for the reversible condensation of dihydroxyacetone phosphate (DHAP) with glyceraldehyde-3-phosphate (G-3P) into fructose-1,6-bisphosphate. 

## Experimental Procedures

### Animals

FynKO mice (*pp59Fyn*) and controls (129SvJ) were obtained from The Jackson Laboratory (Bar Harbor, ME, USA) and housed in a facility equipped with a 12 h light/dark cycle. Animals were fed a standard chow diet (65% carbohydrates, 11% fat, 24% protein (Kcal)). All experiments were performed using 8 to 12 weeks old FynKO mice and age-matched control mice. All experiments were performed in accordance with the recommendations in the Guide for the Care and Use of Laboratory Animals of the National Institutes of Health and approved by the Albert Einstein College of Medicine Institutional Animal Care and Use Committee (IACUC).

### Pyruvate, lactate, glycerol and fructose tolerance tests

Animals were fasted for 16 h and given an intraperitoneal injection of the different substrates in saline (1g/kg). Blood was collected from the lateral vein of the tail prior to and at the indicated times after the injection. Glucose was measured using a Precision Q.I.D glucometer (MediSense, Abbott Laboratories, Abbott Park, IL, USA). 

### Stable isotope assessment of hepatic glucose production

Fasting of animals was initiated at 4:00 P.M and mini-osmotic pumps containing 0.33 mg/µl [U-^13^C_6_] glucose were quickly inserted into the interscapular region of each animal at 7:00 P.M. Animals were sacrificed at 8:00A.M the following morning and plasma were collected. Hepatic glucose production rate was determined using the following equation: HGP (mg[body weight]^-1^ min^-1^)= infusion rate x (1/E^glu^_tracer_-1) where E^glu^_tracer_ is the enrichment of plasma [U-^13^C_6_] glucose (M_6_) determined by GC/MS analysis. In the basal state, HGP equals basal glucose disposal, with clearance defined as (HGP/[basal glucose]). The infusion rate (mg/kg/min) was: mini-osmotic pump rate (8 µl/h calibrated by the manufacturer) x [U-^13^C_6_] glucose concentration (0.25mg/µl)/mouse body weight (kg). GS/MS conditions, sample preparations and a detailed description of equations and calculation for hepatic glucose production can be found in [9,10].

### Glycerol-stimulated glucose production

Fasting of the animals was initiated at 4:00 P.M. At 07:00 P.M, animals were anesthetized under 5% isoflurane and a pre-activated Alzet mini-osmotic pump (Direct Corporation, Cupertino, CA, USA) containing 0.3 mg/µl [U-^13^C]-glycerol (Isotech, Miamisburg, OH, USA) was inserted subcutaneously into the interscapular region of each mouse. Blood samples were collected by orbital bleeding at 10AM, the following morning. Hepatic glucose production (HGP) from glycerol was determined using the following equations: HGP from glycerol (in milligrams per kilograms per minute) = (glycerol production rate) × (glycerol FRC), where FRC is the fractional contribution of glycerol to hepatic glucose synthesis. Glycerol FRC = (M1/2m1) + (M2/m1), where M1 is the enrichment of plasma ^13^C-glucose and M2 the enrichment of plasma ^13^C_2_-glucose, which were synthesized from infused [U-^13^C] glycerol, and m1 is the enrichment of plasma [U-^13^C] glycerol. The enrichments of plasma M1 and M2 glucose, as well as plasma m1 glycerol, were determined by GC/MS [9].

### Tissue sample collection and processing for metabolites analysis

Animals were fasted for 16 hours and then sacrificed. The liver was rapidly dissected and immediately freeze-clamped in liquid nitrogen. Small pieces of 50-100 mg were cut while maintaining the sample frozen in liquid nitrogen, and each piece was accurately weighed. Frozen liver pieces were homogenized in a 1:3 water-chloroform in a glass Dounce (30 strokes of the pestle). Homogenates were centrifuged at 20,000 xg at 4°C for 5 min and the aqueous phases were transferred to auto-sampler vials and analyzed by LC−MS.

### Analysis of hydrophilic metabolites

Analysis of hydrophilic metabolites were carried out by HPLC separations performed in a Waters Acquity UPLC system (Waters Corp., Milford, MA), with a flow rate of 0.2 mL/min for a Acquity UPLC HSS T3, 2.1 x 100 mm, 1.8 µm particle size column (Waters Corp., Milford, MA), using a linear gradient of 0−27% B over 8.0 min (A: 0.1% formic acid in water, ~pH 3.0; B: 100% acetonitrile). Metabolites were detected using a Xevo Triple Quadrupole mass spectrometer (Waters Corp., Milford, MA) equipped with an electrospray ionization source (ESI) operating simultaneously in positive and negative ionization modes, and the data collection was performed by Multiple Reaction Monitoring (MRM) simultaneous screening of the parent and daughter ions [11]. The desolvation gas flow was 900 L/h at a temperature of 500°C, the cone gas flow was 50 L/h and the source temperature at 150 °C. The capillary voltage was 3000 V for positive ion mode; 2800 V for negative ion mode and the cone voltage was modulated by the software depending on each specific MRM metabolite. The dwell times were automatically adjusted by the MassLynx software, based on the number of metabolites being determined at a given time, and by default a minimum of 12 point per peaks were acquired. A volume of 10 µL from of each sample extract was injected and the area under of the peak from each metabolite was used to calculate the concentration with the MassLynx’s software tool TargetLynx (Waters Corp., Milford, MA). Metabolites concentrations were normalized by the amount of tissue used for the extraction, and quantifications are expressed in nmol/mg or ng/mg of liver. 

### Primary hepatocyte isolation and glucose assay

Mouse hepatocytes were isolated from the liver of 8-week old mice by a modified two-step collagenase perfusion protocol [12]. Hepatocytes were plated (10^6^ cells/well in 6-well plates) on collagen-1 coated dishes in DMEM/F12 medium supplemented with dexamethasone (200 nM) and allowed to attach for 12 hours. Cells were starved for 6 hours in glucose and phenol free DMEM medium before being incubated with lactate (20 mM) and pyruvate (2 mM) or glycerol (40 mM) in presence of dexamethasone for 5 hours. Glucose released in the media was quantified using the Glucose Assay kit from Biovision (Milpitas, CA, USA) and normalized by the amount of protein.

### Western blot analysis

Animals were fasted for 16 hours and livers were quickly harvested and flash-frozen in liquid nitrogen. Tissues were homogenized using a BulletBlender (Next Advance, NY, USA) in ice-cold ProteoJETTM Mammalian Cell Lysis Reagent (Fermentas, Glen Burnie, MD, USA) supplemented with protease and phosphatase inhibitors (EMD Chemicals. Inc, Billerica, MA, USA) (Sigma, Saint Louis, MO, USA). Homogenates were centrifuged for 30 min at 12,000 xg at 4°C, and supernatants were collected. Protein concentration was measured using a BCA Protein Assay (Thermo Scientific, Rockford, IL, USA). Proteins samples (20-40 μg) were separated on 10% reducing polyacrylamide gels and electroblotted onto Immobilon-P polyvinylidene difluoride membranes. Immunoblots were blocked with 5% non-fat dry milk in Tris-buffered saline and 0.05% Tween 20 (TBST) for 60 min at room temperature and incubated overnight at 4°C with the indicated antibodies in TBST containing 1% BSA. Blots were washed in TBST and incubated with horseradish peroxidase-conjugated secondary antibodies (1:30,000) for 30 min at room temperature. Membranes were washed in TBST, and antigen-antibody complexes were visualized by chemiluminescence using an ECL kit (Pierce, Rockford, IL, USA). Primary antibodies used were: glycerol kinase (Santa Cruz Biotechnology, Inc., Santa Cruz, CA, USA), aldolase (Cell signalling, Danvers, MA, USA). p115 (BD Biosciences, San Jose, CA, USA) was used as loading control.

### RNA extraction and expression

Mice were fasted for 16 h and sacrificed by cervical dislocation. Livers were collected, immediately homogenized in QIAzol Lysis Reagent (Quiagen, Valencia, CA, USA) using 0.1 mm RNase-free glass beads (Next Advance, Averill Park, NY, USA) in a Bullet Blender (Next Advance Inc, Averill Park, NY, USA). Total RNA was isolated using RNeasy® Mini Kit (Qiagen Sciences, Valencia, CA, USA) and reverse-transcribed to cDNA using the SuperScript VILO cDNA synthesis kit (Invitrogen, Carlsbad, CA, USA). RT-PCR was performed for measurement of mRNA for the *aldolase*, *A*, *B* and C. Relative expression levels of the mRNAs were determined using standard curves. Samples were adjusted for total mRNA content by comparison with *gapdh* expression. 

### Statistics

Results are expressed as mean ± s.e.m and statistical significant (p<0.05) determined by two-tailed Student T-test.

## Results

**Figure 1:**
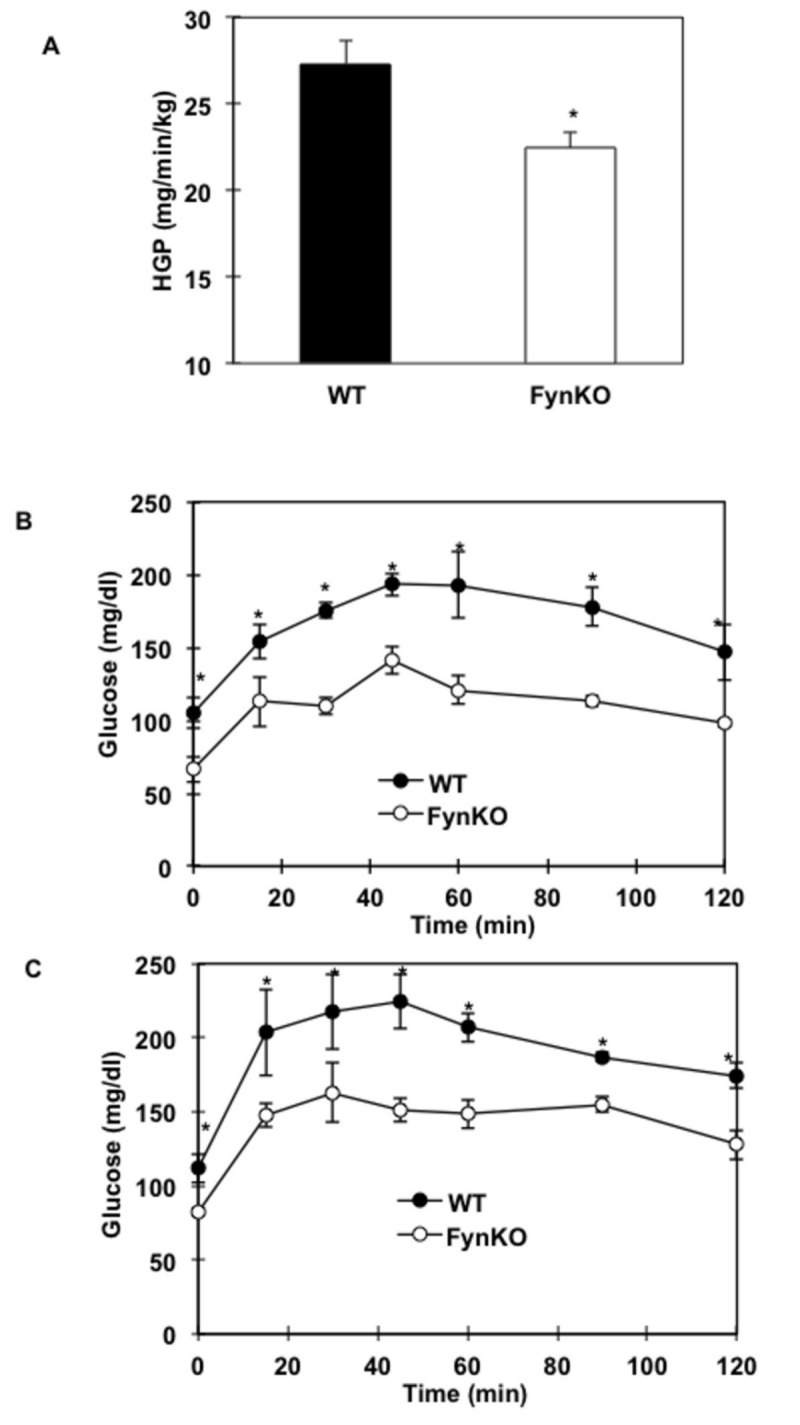
Gluconeogenic capacity assessed by stable isotope assessment of hepatic glucose production (HGP) (A) and intraperitoneal (B) Pyruvate and (C) Lactate;Pyruvate (9:1) tolerance tests in fasted wild type (WT, black circles) and FynKO (open circles) mice. *p<0.05. n= 5 WT, n=5 FynKO, experiments were repeated 4 times for the intraperitoneal tests. *p<0.05. n= 5 WT, n=5 FynKO for the stable isotope experiment.

As previously reported, following an overnight fast FynKO mice display relative hypoglycaemia compared to wild type control mice (Figure 1B, C), suggesting that hepatic *de novo* glucose production was not compensating for the higher glucose disposal observed in these mice. Direct assessment of hepatic glucose production using stable radioisotope [U-^13^C_6_] glucose demonstrated that hepatic glucose production was lowered in the FynKO mice after a fasting period (Figure 1A). To investigate whether FynKO mice were able to use gluconeogenic substrates, pyruvate and lactate tolerance tests were performed. As expected, intraperitoneal injection of pyruvate (pyruvate tolerance test) in control wild type mice resulted in a time-dependent increase in plasma glucose that subsequently recovered back towards steady state levels. However, the relative increase in plasma glucose was significantly reduced in the FynKO mice compared to wild type controls (Figure 1B). Similarly, injection of a combination of lactate and pyruvate also resulted in a diminution of glucose excursion in the FynKO mice compared to wild type mice (Figure 1C). 

**Figure 2:**
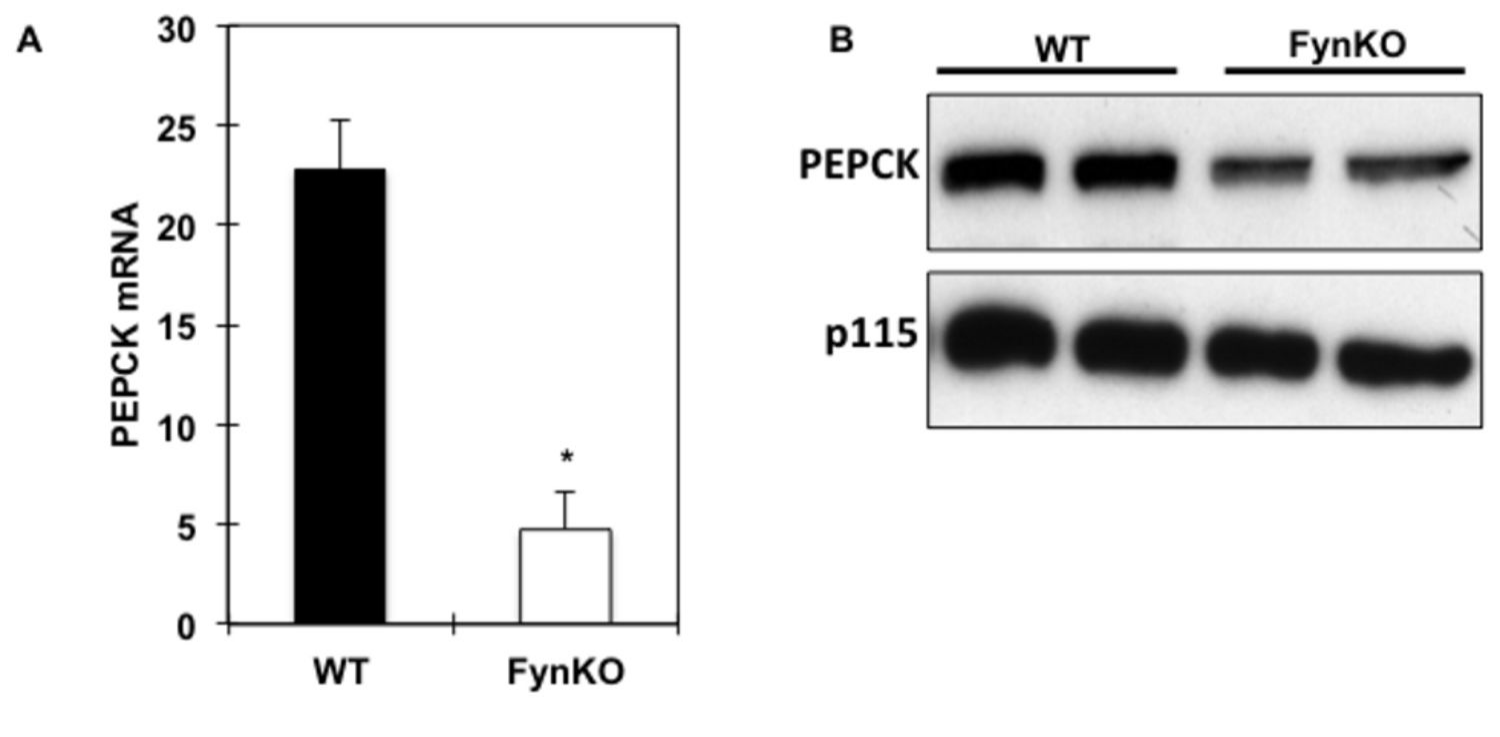
PEPCK activity in the liver of 16-hour fasted wild type (WT) and FynKO mice. PEPCK mRNA expression levels in wild type (WT, black bars) and FynKO (open bars) mice. *p<0.05. n= 5 WT, n=5 FynKO, experiments were repeated 3 times. (B) PEPCK protein levels in wild type (WT) and FynKO mice (blots are representative of n=3 independent experiments).

**Figure 3:**
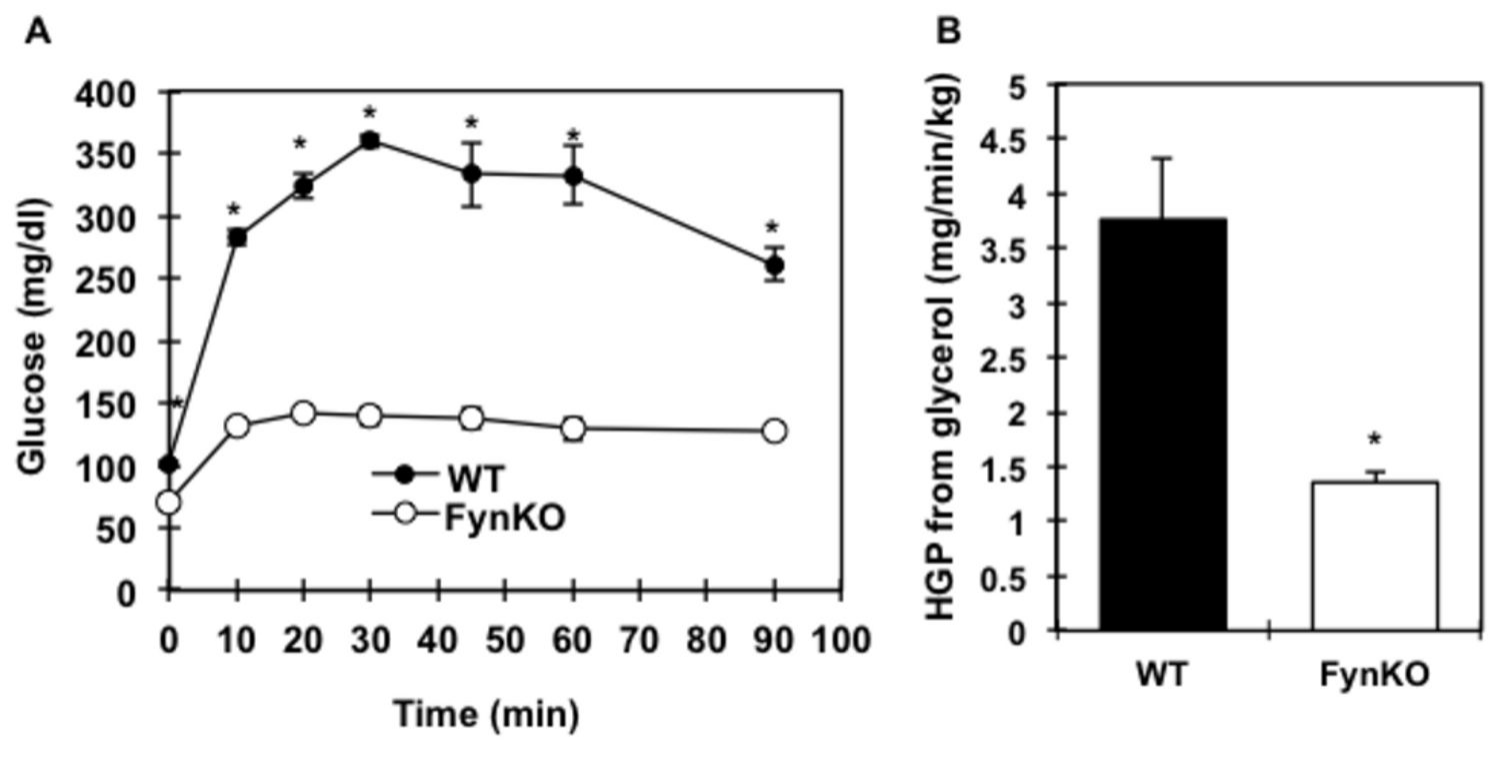
Assessment of glycerol-driven hepatic glucose production in fasted wild type (WT, black circles or black bars) and FynKO (open circles or open bars) mice. (A) intraperitoneal glycerol tolerance test (n=5 animals for each strain), (B) glucose production from [U^13^C] glycerol. *p<0.*05*. n=4 WT, n=4 FynKO.

**Figure 4:**
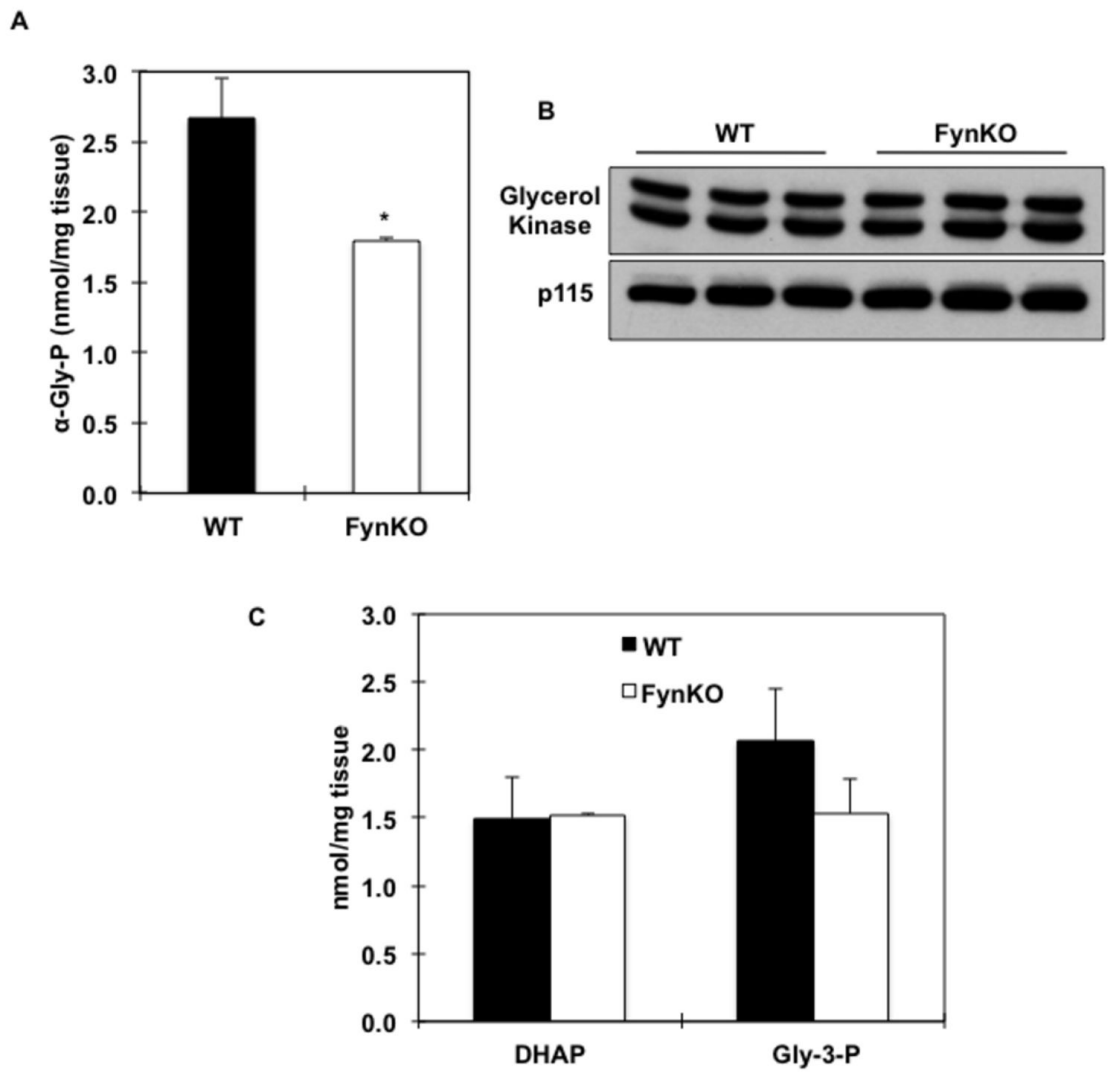
Glycerol metabolites and triose phosphate levels in the liver of wild type (WT, black bars) and FynKO (open bars) mice. (A) alpha-Glycerol phosphate (α-Gly-P) in liver extracts. (B) Glycerol kinase protein expression in the liver of wild type (WT) and FynKO mice. p115 was used as internal loading control. Blot is representative of 3 independent experiments. (C) dihydroxyacetone phosphate (DHAP) and glyceraldehyde-3 phosphate (G-3P) levels in liver extracts. n=3 WT, n=3 FynKO.

In accordance with the relative fasting hypoglycaemia in the FynKO mice, a 5-fold reduction in PEPCK mRNA levels as well as in PEPCK protein expression were found in the fasted liver of the FynKO mice (Figure 2A, B). Since hepatic gluconeogenesis can also occur using glycerol as a substrate thereby bypassing PEPCK, we examined glycerol-driven *de novo* glucose production in the FynKO mice. Whilst intraperitoneal injection of glycerol in control mice resulted in a typical increase and subsequent recovery of plasma glucose levels, this process was markedly blunted in the FynKO mice (Figure 3A). In addition, ^13^C-glycerol incorporation into glucose (Figure 3B) was 3-fold decreased in the FynKO mice, demonstrating that glucose production from glycerol was impaired. Consistent with these *in vivo* data, glucose production from pyruvate, lactate and glycerol was also reduced in primary hepatocytes from FynKO mice (Figure S1). Remarkably, after a 16-hour fasting period, α-glycerol phosphate levels were decreased in the liver of the FynKO mice (Figure 4A) but glycerol kinase expression was unchanged (Figure 4B). Glycerol contributes to gluconeogenesis via its entrance to the triose phosphate pool, i.e., dihydroxyacetone phosphate (DHAP) and glyceraldehyde 3-phosphate (G-3P). However, hepatic DHAP and G-3P levels were normal (Figure 4C), thus suggesting that the reduced gluconeogenesis from pyruvate and lactate was not the result of decreased steady-state levels of triose phosphate in the FynKO liver. 

**Figure 5:**
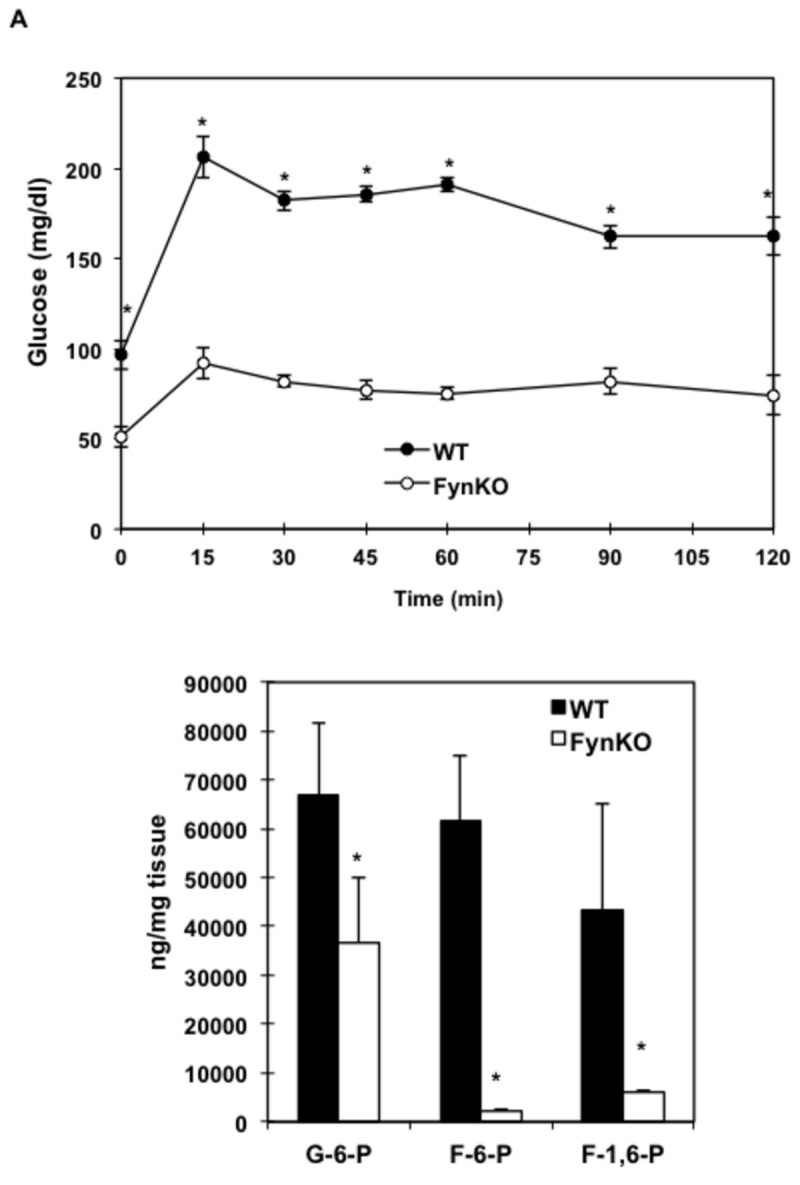
Fructose-driven glucose production in 16-hour fasted wild type (WT) and FynKO mice (A) Fructose tolerance test in fasted wild type (WT, black circles) and FynKO (open circles) mice. *p<0.05. n= 5 WT, n=5 FynKO, experiments were repeated 4 times. (B) Hexose phosphate levels in liver of wild type (WT) and FynKO mice: glucose-6-phosphate (G-6-P), fructose-6-phosphate (F-6-P) and fructose-1,6- bisphosphate (F-1,6-P). *p<0.05, n=3 WT, n=3 FynKO.

**Figure 6:**
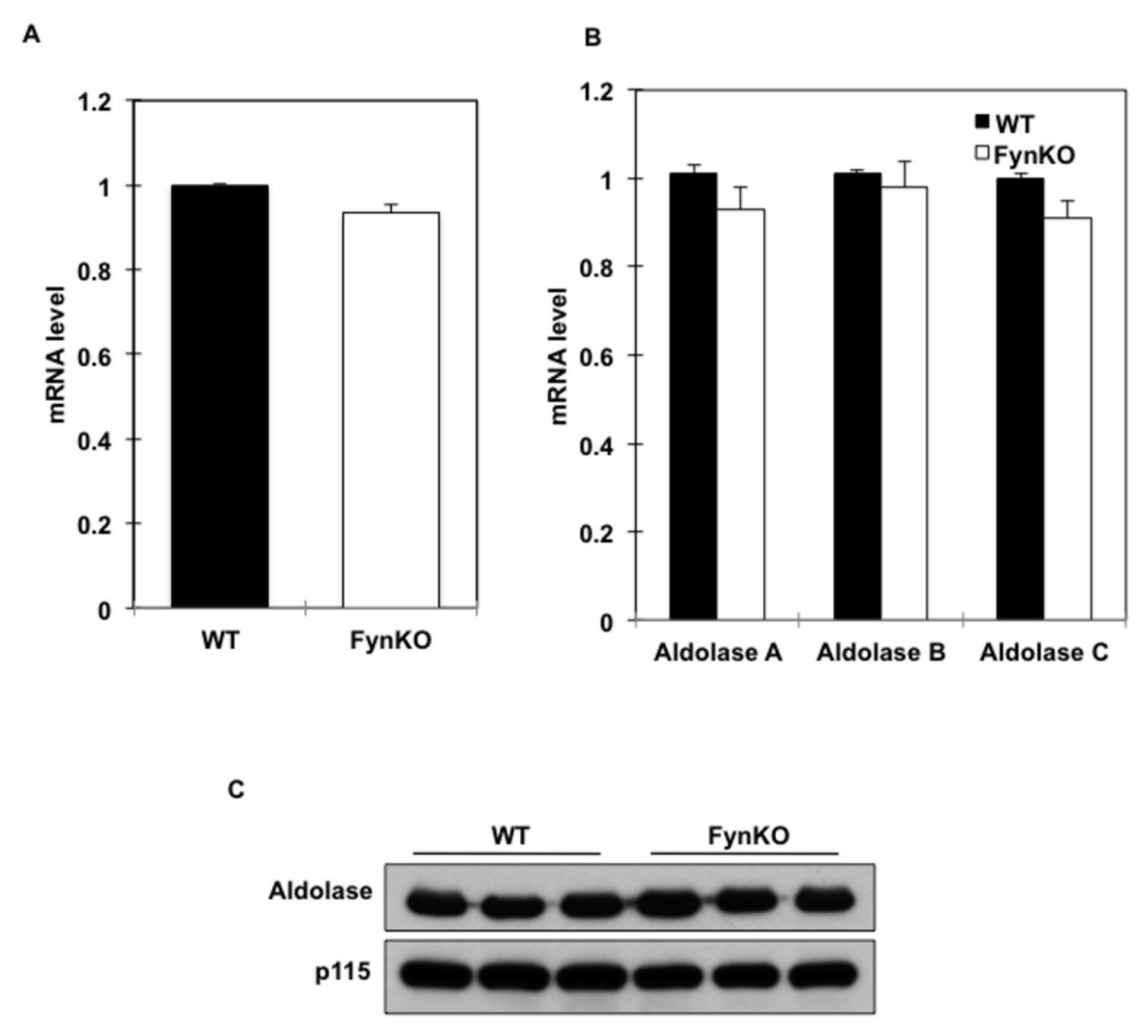
Aldolase and fructokinase expression levels in livers of 16-hour fasted wild type (WT) and FynKO mice. (A) Fructokinase mRNA expression levels and (B) Aldolase A, B and C isoform mRNA expression levels in fasted wild type (WT, black bars) and FynKO (open bars) mice. (C) Aldolase protein expression in the liver of wild type (WT) and FynKO mice. p115 was used as internal loading control. Blots are representative of n=3 independent experiments.

Therefore, we next examined the ability of the liver to produce glucose using fructose as a gluconeogenic substrate. Similarly to the three-carbon substrates (i.e., pyruvate, lactate, glycerol), the FynKO mice were also refractory to glucose production from the six-carbon sugar fructose (Figure 5A), suggesting that fructose was not converted to glucose in the FynKO liver. Consistent with this hypothesis, metabolite analyses demonstrated an approximate 7-fold reduction in the levels of fructose-1,6-bisphosphate and a 30-fold reduction in fructose-6-phosphate levels, with a smaller reduction in glucose-6-phosphate in the liver of the FynKO mice compared to wild type mice (Figure 5B). Interestingly, despite the marked reduction in fructose-1,6-bisphosphate levels, we did not observe significant change in fructokinase gene expression (Figure 6A) or aldolase mRNA expression or aldolase protein levels (Figure 6B, C). 

## Discussion

Recently, we reported that FynKO mice have reduced adiposity, increased energy expenditure and insulin sensitivity that correlated with increased activation of AMP-dependent protein kinase (AMPK) as well as increased fatty acid oxidation in skeletal muscle and adipose tissue but not in the liver [8]. FynKO mice also had reduced glucose excursion during glucose tolerance tests along with fasting hypoglycaemia and low levels of plasma insulin. In general, fasting glucose levels typically reflect the relative contribution of hepatic glucose output and not peripheral tissue insulin sensitivity, as insulin levels are low and glucagon levels are high in the fasted state. Thus, we speculated that Fyn deficiency in the liver functionally impaired *de novo* glucose production from gluconeogenic substrates in the fasted state in a manner that hepatic glucose output is not sufficient to compensate for the increased glucose disposal of these mice, ultimately resulting in lower fasting glucose levels.

The data presented in this study show that the PEPCK pathway, classically considered as the rate-limiting step of the *de novo* hepatic glucose production, is reduced in the liver of the FynKO mice. This is consistent with the fact that FynKO mice have a reduced ability to metabolize three-carbon gluconeogenic substrates, i.e., pyruvate and lactate. However, the FynKO inability to convert glycerol into glucose suggested additional alterations in step (s) that bypass PEPCK. The observation that the triose pool was normal in the fasted liver of the FynKO mice implies that their production is not affected and therefore, the apparent reduction of *de novo* glucose production in the FynKO mice probably results from altered hexose metabolism. In agreement with this prediction, the FynKO mice are both refractory to fructose-driven glucose production and display marked decreases in fructose-1,6-bisphosphate and fructose-6-phosphate levels in the fasted state. 

In the liver, the GLUT2 facilitative transporter is responsible for both glucose and fructose uptake and fructose contributes to *de novo* glucose production via its entrance at the triose step after having been converted into fructose-1-phosphate by fructokinase. Subsequently, fructose-1-phosphate is converted into glyceraldehyde and DHAP in a reversible reaction catalysed by the hepatic isoform of aldolase (aldolase B). Glyceraldehyde is then phosphorylated by the triose kinase and the resulting glyceraldehyde-3-phosphate can either be served as a glycolytic substrate or be condensed with DHAP into fructose-1,6-bisphosphate via the action of the same aldolase to enter the gluconeogenic pathway. The reduced fructose-driven hepatic glucose output could have resulted from a defect in any of these metabolic steps, although we have not observed any changes in fructokinase expression levels. On the other hand, inhibition of triose kinase would not account for the impaired glucose output from other gluconeogenic substrates, i.e., pyruvate, lactate or glycerol and levels of glyceraldehyde-3-phosphate are normal in the FynKO mice. Thus the metabolic step consistent with both the metabolites profile and the tolerance tests (pyruvate, lactate, glycerol and fructose) is the enzymatic step catalysed by aldolase. 

We have been unable to detect any change in aldolase gene or protein expression suggesting that the enzymatic activity of aldolase was compromised. Unfortunately, hepatic aldolase allosteric regulation has not been extensively investigated and remains largely uncharacterized. Biochemical analyses have indicated that aldolase in plants is inhibited by ATP, ADP, AMP and ribose-5-phosphate and that mammalian skeletal muscle aldolase is allosterically inactivated by oxidized gluthathione [13,14]. Skeletal muscle aldolase has also been shown to interact and decrease the inactivation of the enzyme phosphofructokinase [15,16]. This association not only alters the allosteric regulation of phosphofructokinase but also increases the activity of aldolase by approximately 2-fold [17], which may provide an advantage for channelling substrates through the glycolytic pathway. However, skeletal muscle and liver express different aldolase isoforms, aldolase B being preferentially expressed in liver and isoform A in the skeletal muscle [18]. Although the majority of the structure is conserved between these isoforms, variations in isozyme-specific regions appear to play a significant role in the substrate preference of each isoform [19]. Therefore, whether the regulatory properties of aldolase in skeletal muscle also occur in the liver *in vivo* remain to be clarified. Additionally, regulation of the reverse reaction, i.e., formation of fructose-1,6-bisphosphate from the condensation of DHAP with G-3P has not been delineated. The metabolite profiles (decreased fructose-1,6-bisphosphate and fructose-6-phosphate levels) as well as the impaired response to gluconeogenic substrates bypassing the PEPCK step would logically suggest a reduction of the aldolase enzymatic activity in the reverse reaction, that is a decrease in DHAP and G-3P condensation. However, in this case, it would be expected that reduced aldolase activity would result in accumulation of these metabolites, whose levels were, in fact, normal. Therefore, it remains formally possible that the glycolytic reaction (production of DHAP and G-3P from fructose-1,6-bisphosphate) could be increased, therefore hexose-phosphate levels are decreased as a result of being pushed into the triose pool in lieu of serving as glucose precursors. In any case, this supports allosteric regulation but since this sort of regulation will be lost during the preparation of liver tissue extracts, it is therefore not possible to demonstrate this fact using *in vitro* enzymatic assays.

In summary, our data demonstrate a marked reduction in gluconeogenic substrate-driven glucose production in the liver of the FynKO mice, which functions in metabolic concert with enhanced peripheral tissue insulin sensitivity and fatty acid oxidation and results in fasting hypoglycaemia. The demonstration that lack of Fyn kinase in the liver affects *de novo* gluconeogenesis further supports the development of specific inhibitors of Fyn kinase activity that have been shown to effectively increase energy expenditure and adipose mass selective weigh loss in normal wild type mice [8,20]. Future studies are now needed to identify the allosteric regulators of aldolase B in the liver and to determine how Fyn kinase regulates their levels along with aldolase B activity. 

## Supporting Information

Figure S1 **Lactate/pyruvate and glycerol-driven glucose production is reduced in isolated FynKO primary hepatocytes.** Glucose released in the culture media of wild type (WT- black bars) and FynKO (open bars) primary hepatocytes incubated with lactate and pyruvate or with glycerol for 5 hours. *p<0.05, n= 3 independent experiments.
(TIFF)
