# Supplementary figures and images for: Alteration of De Novo Glucose Production Contributes to Fasting Hypoglycaemia in Fyn Deficient Mice
Source: PLoS One. 2013 Nov 28;8(11):e81866. doi: 10.1371/journal.pone.0081866 (PMC3842980; doi:10.1371/journal.pone.0081866)

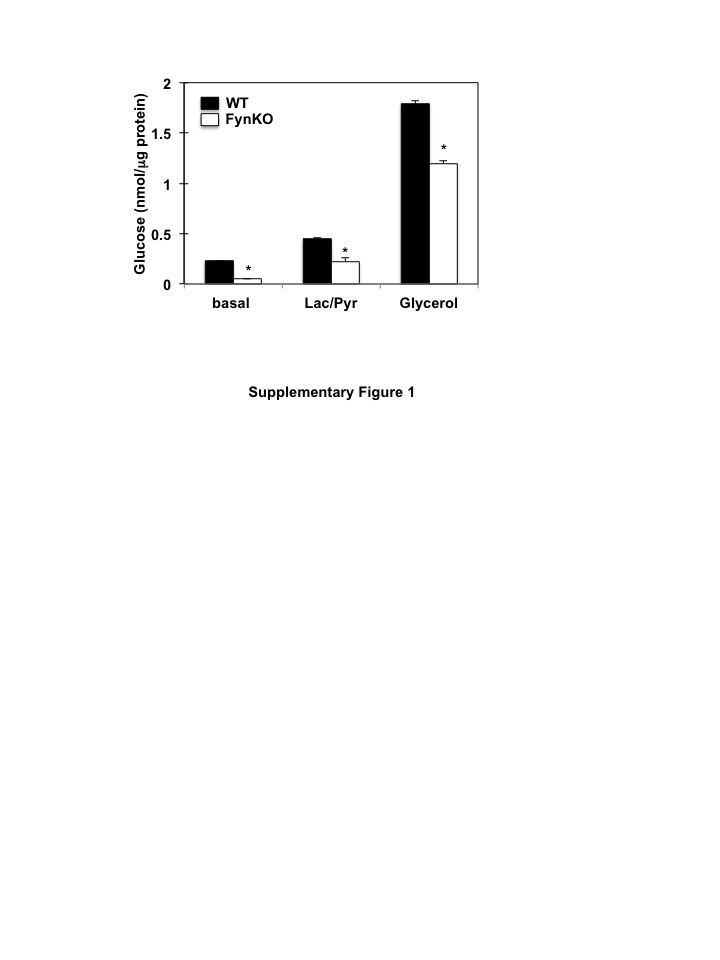

Supplement: Figure S1 — Lactate/pyruvate and glycerol-driven glucose production is reduced in isolated FynKO primary hepatocytes. Glucose released in the culture media of wild type (WT- black bars) and FynKO (open bars) primary hepatocytes incubated with lactate and pyruvate or with glycerol for 5 hours. *p<0.05, n= 3 independent experiments. (TIFF) [file pone.0081866.s001.tiff]
